# Complete Genome Sequence of *Lactobacillus jensenii* Strain SNUV360, a Probiotic for Treatment of Bacterial Vaginosis Isolated from the Vagina of a Healthy Korean Woman

**DOI:** 10.1128/genomeA.01757-16

**Published:** 2017-03-09

**Authors:** Sunghee Lee, Hyun Ju You, Bomi Kwon, GwangPyo Ko

**Affiliations:** a KoBioLabs, Inc., Seoul, Republic of Korea; b N-Bio, Seoul National University, Seoul, Republic of Korea; c Department of Environmental Health Sciences, Graduate School of Public Health, Seoul National University, Seoul, Republic of Korea; d Institute of Health and Environment, Seoul National University, Seoul, Republic of Korea; e Center for Human and Environmental Microbiome, Seoul National University, Seoul, Republic of Korea

## Abstract

*Lactobacillus jensenii* SNUV360 is a potential probiotic strain that shows antimicrobial activity for the treatment of bacterial vaginosis. Here, we present the complete genomic sequence of *L. jensenii* SNUV360, isolated from a vaginal sample from a healthy Korean woman. Analysis of the sequence may provide insight into its functional activity.

## GENOME ANNOUNCEMENT

Bacterial vaginosis (BV) is a common condition associated with numerous adverse health outcomes in women of reproductive age. BV can be characterized by a shift in the vaginal flora from *Lactobacillus* spp. dominance to a more diverse microbial environment (1). Some *Lactobacilllus* spp. strains have been shown to decrease pathogenic bacteria in vaginal environments of women diagnosed with BV (2, 3).

In this study, the strain *Lactobacillus jensenii* SNUV360, isolated from the vaginas of healthy women, shows functional properties in the treatment of vaginal infection caused by lactobacilli deficiency. Here, we present the complete genome sequence of this probiotic strain.

In order to perform the complete genome sequencing of the strain *L. jensenii* SNUV360, high throughput sequencing technology was implemented using the PacBio platform (Pacific Biosciences, Menlo Park, CA). A 20-Kb library was constructed with purified DNA affixed to single-molecule real-time (SMRT) cell and was sequenced using P6-C4 chemistry with a data collection time of 4 h. The sequencing run provided a total of 143,207 reads with a quality score of Q20. The number of bases was 964,237,020 bp. *De novo* assembly employed the default parameters of the Hierarchical Genome Assembly Process approach version 3 (HGAP3) (4). The *L. jensenii* SNUV360 genome consisted of a 1,672,949 bp single-chromosome contig, with coverage of 420× and G+C content of 34.4%. No plasmids were detected.

The assembled genome sequences were annotated using the Prokka annotation pipeline, version 1.11 (5), which predicted tRNA, rRNA, and mRNA genes. Putative gene products were then assigned to protein-coding genes (CDSs) based on their similarity to the sequences in the respective database. Curated virulence factors and antibiotic resistance genes were estimated by using IslandViewer3 (6) against the Virulence Factor Database (VFDB) (7) of virulence factors, and Comprehensive Antibiotic Resistance Database (CARD) (8) of antibiotic resistance genes.

The genome contains 1,595 CDSs, 58 tRNA genes, and 12 rRNA genes. The *L. jensenii* SNUV360 genome was compared with the reference strain *L. jensenii* TL2937 (genome accession number NZ_MDTN01000000) with the Rapid Annotations using the Subsystems Technology (RAST) server (9). In this comparison, we detected 42 elements absent in the published strain *L. jensenii* TL2937. No remarkable antibiotic resistance or virulence-associated genes were found. The analysis of the complete genome of *L. jensenii* SNUV360 may assist in understanding the mechanisms involved in its effect against bacterial vaginosis.

### Accession number(s).

The results of this whole-genome project have been deposited at GenBank under accession no. CP018809.
